# On Modern Progress in Ophthalmic Medicine and Surgery

**Published:** 1894-05-05

**Authors:** Robert Brudenell Carter

**Affiliations:** Consulting Ophthalmic Surgeon to St. George's Hospital


### On Modern Progress in Ophthalmic Medicine and Surgery.

By Robert Brudenell Carter, F.R.C.S., Consulting Ophthalmic Surgeon to St. George's Hospital.

The use of chloroform in the extraction of cataract, once introduced, rapidly gained ground, and, in a large proportion of instances, entirely fulfilled the expectations of its advocates. There was no muscular spasm, and no difficulty either in localising the first incision, or in completing the subsequent steps of the operation. Sometimes, however, the chloroform turned traitor, and when it did so the consequences were frequently disastrous. The stage of partial recovery was sometimes attended by sudden and severe muscular spasm, considerable loss of vitreous, and occasionally attended by the rupture of a retinal vessel, an occurrence which was fatal to every hope of restoration of sight, and which might even justify the removal of the eye before recovery was complete. When there was no spasm there was sometimes severe and long-continued vomiting, by which, after a perfectly satisfactory operation, I have seen the eye-ball absolutely emptied of its contents. Notwithstanding these drawbacks, the balance of advantage was decidedly in favour of chloroform; and spasm or vomiting were regarded as accidents which might occur, but which were to be guarded against as much as possible. Preliminary abstinence from solid food was strictly enforced, and old people were supported under this abstinence by the careful administration of small quantities of beef tea or of alcoholic stimulants. Complete anæsthesia was maintained, not only until the operation was completed, but until the eye was closed and supported by a packing of cotton wool, retained by a firm and carefully-applied bandage. A similar packing, supported by the hand, was used whenever vomiting occurred before the operation was finished. The chloroform had its attendant evils, but these were far less considerable than those which it set aside,

About 1870, the pendulum of surgical opinion, especially in America, began to swing back in the direction of ether, as a less dangerous anæsthetic agent than chloroform. As it was then commonly given in this country, it was not so suitable as chloroform for eye cases; but in 1872, at the International Ophthalmological Congress held in London, Dr. Joy Jeffries, of Boston, U.S.A., read a paper describing a somewhat different method of administration, and at my request he exhibited his method at St. George's Hospital, where, during the Congress, I operated upon a series of patients to whom he had given pure ether, and with whom it succeeded perfectly well. After that I used it, either pure or mixed with chloroform and alcohol, in the great majority of my cases both in public and private practice, being influenced mainly by its actual or supposed greater safety, but always feeling that it was inferior to pure chloroform on account of its tendency to produce congestion of superficial tissues, and thus to entail an undesirable amount of bleeding from the cut iris. Still, on the assumption that ether involved less risk to human life than chloroform, the increased bleeding was not of material importance.

Matters remained in this position until the latter part of 1884. It had long been known to travellers that the mountaineers of the Andes were accustomed to chew the leaves of the Erythroxylon coca, and to derive from them greatly increased power of enduring abstinence from food. Von Humboldt had described their practice in this respect; but the matter remained almost without notice until it was taken up by the late Sir Robert Christison, of Edinburgh, who, about 1860, after reading Humboldt's narrative, obtained a supply of the leaves, and instituted a variety of experiments with them. He appeared to believe, as Humboldt had undoubtedly believed, that the coca contained some sustaining agency, but his experiments were not productive of any important result. He separated the active principle, and called it cocaine, but was unable to obtain evidence of its efficacy. The romance writer, Gustave Aimard, introduced the leaf into some of his stories of Peruvian life; and it was probably this circumstance which induced an enterprising French pharmacist to manufacture "coca wine," and to advertise it as a valuable medicine. The advertisements had at least the merit of calling medical attention to the existence of the drug; and in 1884, Dr. Carl Koller, a young Austrian physician, determined to institute a research into its properties. He was rewarded by the curious discovery that, after he had touched his tongue with a concentrated solution, the spot so touched was rendered for a time numb and insensitive. Experimentation upon animals confirmed this discovery, and showed that cocaine acted as a local anæsthetic, rendering the part to which it was applied insensitive to pain, but in no way affecting the general consciousness. On account of the thinness and moisture of the conjunctiva, and of the structure of the cornea, it was more readily absorbed by the eye than by any other part of the body, and it came at once into use as a local anæsthetic for the purposes of ophthalmic surgery. It became manifest that its supposed "sustaining action" was imaginary, and that its power of enabling those who used it to resist abstinence depended upon its rendering the stomach insensitive to the pains of hunger. It produces considerable contraction of the blood vessels of any part to which it is applied, thus diminishing the bleeding from an incision, and it dilates the pupil, all its effects being very temporary. My own first operation upon the eye by means of its assistance was performed in November, 1884; and, since that time, I have had scarcely any occasion to employ a general anæsthetic. When first introduced, and before experience of its effects had been gained, the anæsthesia produced by cocaine sometimes fell short of what would have been desirable; but surgeons soon learned how to overcome difficulties of this kind, and cataract and other operations are now performed almost without the knowledge of the patient, who, at the same time, is able to assist the operator by turning the eye in any direction that may be required. Muscular spasm is unknown, vomiting is a forgotten danger, the utmost deliberation and care may be exercised by the operator, and the patient may be comforted and assured, before the eye is closed, by being permitted to test vision sufficiently to render him sure that it has been restored. As far as ophthalmic surgery is concerned, the introduction of cocaine, although its use is very limited in other departments, has been of far greater value than the introduction of chloroform.

